# Supplementary material for: Host Cell Response to Rotavirus Infection with Emphasis on Virus–Glycan Interactions, Cholesterol Metabolism, and Innate Immunity
Source: Viruses. 2023 Jun 21;15(7):1406. doi: 10.3390/v15071406 (PMC10385841; doi:10.3390/v15071406)
Supplement: Supplementary file 1 [file viruses-15-01406-s001.zip › viruses-2438202-supplementary.pdf]

Supplementary Materials:

Supplementary Table S1. Summary of DEGs affected by both G9P[13] and G5P[7] strains. Genes are shown with '↓' and'↑' referring to down or upregulation, respectively. These are also depicted with red arrows signifying upregulation, and blue representing down regulation. Differentially expressed genes are bolded. The upregulated/downregulated genes were determined using a cut off ± ≥ 2 or ≤ 2 when respectively compared with the control group (non-treated). All genes listed account for active protein coding.

Gene Symbol	Description	G5P[7]	G9P[13]
**ABCG2**	ATP-binding cassette, sub-family G (WHITE), member 2	↓	↑
**ABCG5**	ATP binding cassette subfamily G member 5, transcript variant X1	↓	↑
**ABCG8**	ATP binding cassette subfamily G member 8, transcript variant X1	↓	↑
**ANPEP**	alanyl aminopeptidase, membrane	↓	↑
**AOC1**	amine oxidase, copper containing 1, transcript variant X5	↓	↑
**BATF3**	basic leucine zipper ATF-like transcription factor 3	↓	↓
**C1H9orf16**	chromosome 1 C9orf16 homolog	↑	↑
**C6H19orf81**	chromosome 6 C19orf81 homolog, transcript variant X1	↑	↑
**C8H4orf48**	chromosome 8 C4orf48 homolog	↑	↑
**CATSPER4**	cation channel sperm associated 4	↓	↑
**CDK5R1**	cyclin dependent kinase 5 regulatory subunit 1	↓	↓
**CFAP61**	cilia and flagella associated protein 61, transcript variant X3	↓	↓
**CLIC5**	chloride intracellular channel 5	↓	↑
**CMC4**	C-X9-C motif containing 4, transcript variant X1	↑	↑
**CNTNAP2**	contactin associated protein-like 2	↓	↑
**CORO2B**	coronin 2B, transcript variant X2	↓	↓
**CPA6**	carboxypeptidase A6, transcript variant X1	↓	↓
**CRTAC1**	cartilage acidic protein 1, transcript variant X2	↑	↑
**CSDC2**	cold shock domain containing C2	↓	↑
**CYP26A1**	cytochrome P450, family 26, subfamily A, polypeptide 1	↓	↓
**CYS1**	cystin 1	↑	↑
**DCST1**	DC-STAMP domain containing 1	↓	↑
**DENND1C**	DENN domain containing 1C	↓	↓
**DENND2A**	DENN domain containing 2A, transcript variant X5	↓	↓
**DPCR1**	diffuse panbronchiolitis critical region 1	↓	↑
**EGR2**	early growth response 2	↓	↓
**ELMOD1**	ELMO domain containing 1, transcript variant X4	↓	↓
**ENKD1**	enkurin domain containing 1, transcript variant X1	↑	↑
**FAM129C**	family with sequence similarity 129 member C	↑	↑
**FAM25A**	family with sequence similarity 25 member A	↑	↑
**FAM71E1**	family with sequence similarity 71 member E1, transcript variant X2	↑	↑
**FAR2**	fatty acyl-CoA reductase 2, transcript variant X1	↓	↑
**FGF22**	fibroblast growth factor 22	↑	↑
**FOSB**	FosB proto-oncogene, AP-1 transcription factor subunit	↑	↑
**FOXH1**	forkhead box H1, transcript variant X3	↑	↑
**GAREM2**	GRB2 associated regulator of MAPK1 subtype 2, transcript variant X2	↓	↓
**GGN**	gametogenetin	↑	↑

**GGT5**	gamma-glutamyltransferase 5, transcript variant X2	↑	↑
**GPAT2**	glycerol-3-phosphate acyltransferase 2, mitochondrial, transcript variant X3	↑	↑
**GPR156**	G protein-coupled receptor 156, transcript variant X6	↓	↓
**HDAC9**	histone deacetylase 9, transcript variant X22	↓	↓
**HHLA2**	HERV-H LTR-associating 2, transcript variant X22	↓	↑
**HMGCS2**	3-hydroxy-3-methylglutaryl-CoA synthase 2	↓	↑
**IFIT1**	interferon-induced protein with tetratricopeptide repeats 1	↓	↓
**IGFBP3**	insulin like growth factor binding protein 3	↓	↓
**JPH1**	junctophilin 1, transcript variant X1	↓	↓
**KIF1A**	kinesin family member 1A	↓	↑
**LEAP2**	liver enriched antimicrobial peptide 2	↑	↑
**LEKR1**	leucine, glutamate and lysine rich 1, transcript variant X1	↑	↑
**LRRC23**	leucine rich repeat containing 23, transcript variant X2	↑	↑
**LRRC31**	leucine rich repeat containing 31, transcript variant X2	↑	↑
**LTB4R**	leukotriene B4 receptor, transcript variant X1	↓	↑
**LTF**	lactotransferrin	↓	↓
**MAF**	MAF bZIP transcription factor, transcript variant X1	↓	↑
**MCAM**	melanoma cell adhesion molecule, transcript variant X3	↓	↓
**MDFI**	MyoD family inhibitor, transcript variant X4	↓	↓
**MIA**	melanoma inhibitory activity, transcript variant X1	↓	↓
**MIR7138**	ssc-miR-7138-3p	↓	↓
**MOGAT2**	monoacylglycerol O-acyltransferase 2	↓	↑
**MRC1**	mannose receptor C-type 1	↓	↑
**MTERF2**	mitochondrial transcription termination factor 2	↓	↓
**MTTP**	microsomal triglyceride transfer protein	↓	↑
**MUC12**	mucin 12, cell surface associated	↓	↑
**MYH15**	myosin heavy chain 15	↑	↓
**NAGS**	N-acetylglutamate synthase	↓	↑
**NEURL3**	neuralized E3 ubiquitin protein ligase 3	↑	↑
**NKX3-2**	NK3 homeobox 2	↓	↓
**OASL**	2'-5'-oligoadenylate synthetase like	↑	↑
**ONECUT2**	one cut homeobox 2	↑	↑
**OVGP1**	oviductal glycoprotein 1	↓	↓
**PCDH7**	protocadherin 7	↓	↓
**PDGFRL**	platelet derived growth factor receptor like, transcript variant X1	↓	↓
**PECAM1**	platelet and endothelial cell adhesion molecule 1	↓	↓
**PIK3CG**	phosphatidylinositol-4,5-bisphosphate 3-kinase catalytic subunit gamma	↓	↓
**PIRT**	phosphoinositide interacting regulator of transient receptor potential channels, transcript variant X2	↑	↑
**PLBD1**	phospholipase B domain containing 1	↓	↓
**PLPPR2**	phospholipid phosphatase related 2, transcript variant X1	↑	↑
**PPP1R36**	protein phosphatase 1 regulatory subunit 36, transcript variant X1	↓	↓
**RAB39A**	RAB39A, member RAS oncogene family, transcript variant X1	↓	↓
**RAB4B**	RAB4B, member RAS oncogene family, transcript variant X1	↑	↑
**RET**	ret proto-oncogene	↓	↓

**RGL1**	ral guanine nucleotide dissociation stimulator like 1, transcript variant X2	↓	↓
**RGS16**	regulator of G protein signaling 16	↑	↑
**RLN3**	relaxin 3	↑	↑
**ROM1**	retinal outer segment membrane protein 1	↑	↑
**RSPH1**	radial spoke head 1 homolog, transcript variant X3	↓	↓
**RSPH9**	radial spoke head 9 homolog, transcript variant X2	↑	↑
**RTN4RL1**	reticulon 4 receptor like 1, transcript variant X1	↓	↓
**SCNN1A**	sodium channel epithelial 1 alpha subunit	↑	↑
**SECTM1**	secreted and transmembrane 1	↑	↑
**SLA-5**	MHC class I antigen 5	↑	↑
**SLC10A4**	solute carrier family 10 member 4	↓	↓
**SLC26A7**	solute carrier family 26 member 7, transcript variant X5	↑	↑
**SLC30A10**	solute carrier family 30 member 10	↑	↑
**SLC35G2**	solute carrier family 35 member G2, transcript variant X1	↓	↓
**SLC51B**	solute carrier family 51 beta subunit, transcript variant X1	↓	↑
**SLC6A4**	solute carrier family 6 member 4, transcript variant X1	↓	↑
**SNAI1**	snail family transcriptional repressor 1	↑	↑
**SNX22**	sorting nexin 22, transcript variant X2	↓	↓
**SPDEF**	SAM pointed domain containing ets transcription factor	↑	↑
**ST18**	ST18, C2H2C-type zinc finger, transcript variant X18	↓	↓
**STAG3**	stromal antigen 3, transcript variant X2	↑	↑
**STARD7**	StAR related lipid transfer domain containing 7	↓	↓
**SYBU**	syntabulin	↑	↑
**SYT12**	synaptotagmin 12, transcript variant X4	↑	↑
**TEX14**	testis expressed 14, intercellular bridge forming factor	↓	↓
**TFF2**	trefoil factor 2	↑	↑
**TGM3**	transglutaminase 3	↑	↑
**TMCO2**	transmembrane and coiled-coil domains 2	↑	↑
**TMEM255B**	transmembrane protein 255B, transcript variant X2	↑	↑
**TMEM88B**	transmembrane protein 88B	↑	↑
**TREX2**	three prime repair exonuclease 2, transcript variant X2	↓	↑
**TRIM31**	tripartite motif containing 31	↓	↑
**TUBAL3**	tubulin alpha like 3	↓	↑
**WFIKKN1**	WAP, follistatin/kazal, immunoglobulin, kunitz and netrin domain containing 1	↑	↑
